# Supplementary material for: The interactome of intact mitochondria by cross-linking mass spectrometry provides evidence for coexisting respiratory supercomplexes
Source: Mol Cell Proteomics. 2017 Dec 8;17(2):216–32. doi: 10.1074/mcp.RA117.000470 (PMC5795388; doi:10.1074/mcp.RA117.000470)
Supplement: Supplemental Data [file supp_RA117.000470_133922_0_supp_23978_fzffwf.zip › spectra_annotation/mito_DR_spectra_annotation/275-1-13-1-5-1.pdf]

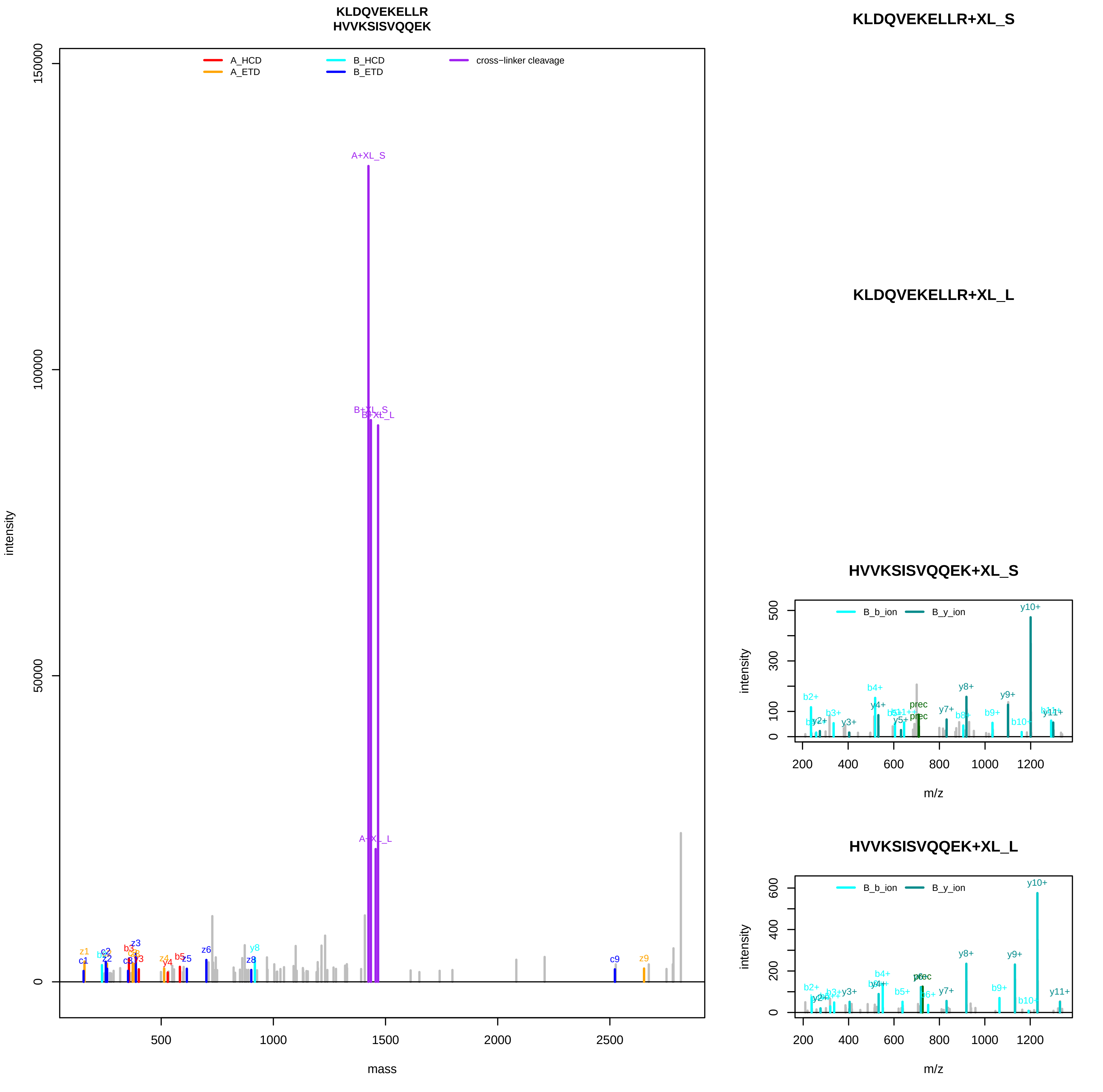
KLDQVEKELLR
HVVKSISVQQEK
A_HCD
A_ETD
B_HCD
B_ETD
cross−linker cleavage
A+XL_S
B+XL_S
B+XL_L
A+XL_L
intensity
mass
0
50000
100000
150000
500
1000
1500
2000
2500
z1
c1
c2
z2
b3
z3
c3
y3
z4
y4
b5
z5
z6
y8
z8
c9
z9
KLDQVEKELLR+XL_S
KLDQVEKELLR+XL_L
HVVKSISVQQEK+XL_S
B_b_ion
B_y_ion
y10+
b2+
y2+
b3+
y3+
b4+
y4+
y5+
prec
y7+
y8+
b8+
y9+
b9+
b10+
y11+
intensity
m/z
HVVKSISVQQEK+XL_L
B_b_ion
B_y_ion
y10+
b2+
y2+
b3+
y3+
b4+
y4+
b5+
prec
y6+
y7+
y8+
y9+
b9+
b10+
y11+
intensity
m/z
